# How to demonstrate factors associated with peri‐esophageal vagal nerve injury during catheter ablation for atrial fibrillation

**DOI:** 10.1002/joa3.13048

**Published:** 2024-04-28

**Authors:** Naoya Kataoka, Teruhiko Imamura

**Affiliations:** ^1^ Second Department of Internal Medicine University of Toyama Toyama Japan

To editor:

Peri‐esophageal vagal nerve injury (PNI) can unpredictably occur during atrial fibrillation ablation procedures. Yoshimura and colleagues have demonstrated an association between symptomatic PNI and high contact force near the esophagus.^1^ However, several concerns have been raised.

While the authors identify contact force as a major cause of PNI,^1^ other factors such as baseline impedance and the rate of rise of esophageal temperature during ablation have also been proposed.^2^, ^3^

The authors limited ablation power to <30 W and ablation duration to within 30 s, irrespective of the ablation index, when ablating the left atrial posterior wall near the esophagus.^1^ Ablation was terminated promptly if the esophageal temperature reached 40°C. Nevertheless, recent literature indicates that high‐power short‐duration ablation may offer advantages over moderate‐power moderate‐duration ablation, including improved durability of ablation, reduced procedure time, and decreased risk of irreversible tissue injury.^4^ Additionally, concerns persist regarding the optimal placement of ablation lines on the left atrial posterior wall to prevent PNI.

In the current era, moderate‐power ablation, as employed by the authors, is seldom the initial choice. Instead, cryoballoon and pulsed‐field ablations are preferred. How do the authors' findings translate to contemporary procedures?

## FUNDING INFORMATION

None.

## CONFLICT OF INTEREST STATEMENT

Authors declare no conflict of interests for this article.

## ETHICS APPROVAL

None.

## Data Availability

This manuscript does not contain any data to be shared.
